# Effect of rapid rehabilitation surgery on postoperative rehabilitation, complications and long-term prognosis in radical laryngectomy: a PSM matching study

**DOI:** 10.3389/fmed.2025.1492210

**Published:** 2025-02-19

**Authors:** Yibo Huang, Meifang Wang, Jianfeng Pu, Yunpeng Zang, Teng He

**Affiliations:** ^1^Department of Anesthesiology, Affiliated Changshu Hospital of Nantong University, Changshu, Jiangsu Province, China; ^2^Department of Otorhinolaryngology, The Affiliated Hospital of Xuzhou Medical University, Xuzhou, Jiangsu Province, China; ^3^Department of Otorhinolaryngology, Affiliated Changshu Hospital of Nantong University, Changshu, Jiangsu Province, China

**Keywords:** rapid rehabilitation surgery, radical laryngectomy, postoperative rehabilitation, long-term prognosis, PSM matching

## Abstract

**Objective:**

To evaluate the effects of Enhanced Recovery After Surgery (ERAS) on postoperative rehabilitation, complications, and long-term prognosis in patients undergoing radical laryngectomy using a single-center propensity score matching (PSM) study.

**Methods:**

A retrospective cohort study included patients newly diagnosed with laryngeal cancer between January 1, 2019, and January 1, 2021, scheduled for partial laryngectomy. The control group (CG) comprised patients treated with standard interventions in 2019, while the research group (RG) included patients undergoing ERAS in 2020. After exclusions, 233 individuals remained: 94 in the RG and 204 in the CG. Following PSM in a 2:1 ratio, there were 180 in the CG and 90 in the RG. Relevant indices were analyzed.

**Results:**

No significant differences were found in baseline characteristics (*p* > 0.05). The RG showed significantly lower hospital stay, nasogastric tube and tracheal cannula duration, early enteral nutrition, hospitalization expenses, and readmission rates compared to the CG (*p* < 0.05). The RG had higher albumin and prealbumin levels on postoperative days 3 and 7 (*p* < 0.05) but not on day 1 (*p* > 0.05). No significant differences were found in 1-year or 2-year overall survival rates, nor in recurrence-free survival rates between the groups (*p* > 0.05), though the RG showed marginally better survival.

**Conclusion:**

ERAS treatment for postoperative laryngeal cancer patients reduces hospitalization duration, nasogastric tube and tracheal cannula use, costs, readmission rates, and complications, while accelerating recovery and facilitating early discharge. ERAS enhances patient comfort and clinical outcomes, supporting broader clinical adoption.

## Introduction

Laryngeal cancer is a common malignant tumor in otolaryngology surgery, and its incidence accounts for about 10–20% of otolaryngology malignant tumors (1). The incidence of dysphagia following partial laryngectomy ranges widely from 11.9 to 72.1% (2). Partial supracricoid laryngectomy is highly recommended for preserving laryngeal integrity, swallowing function, and vocal capabilities. However, in the early postoperative period, the incidence of dysphagia can be as high as 100%. Dysphagia not only contributes to malnutrition and delayed wound healing but also poses risks such as aspiration and chronic lung infections. These complications significantly impact patients’ quality of life and their physical and mental health, leading to prolonged hospital stays and increased utilization of healthcare resources (3). Even about 10–15% of postoperative patients with laryngeal cancer will eventually die due to hidden aspiration (4). Accelerated rehabilitation surgery is a new concept popular at home and abroad at this stage. Enhanced Recovery After Surgery (ERAS) is a term coined by Professor Kehlet of Denmark in 1997 to describe patient-centered, evidence-based, multidisciplinary team developed pathways for a surgical specialty and facility culture to minimize the patient’s surgical stress response, maximize their physiologic function, and speed up recovery. Collaboration among various disciplines including nurses, anesthesiologists, nutritionists, and rehabilitators is crucial in the perioperative care of patients. While surgeons play a pivotal role, the involvement of these other healthcare professionals is equally essential (5). Currently, Enhanced Recovery After Surgery (ERAS) research is most advanced in colorectal cancer, with other fields such as obstetrics and gynecology, thoracic surgery, and hepatobiliary surgery also adopting the concept of accelerated rehabilitation surgery. But still, few clinical research have been conducted on the application of ERAS in the perioperative period of laryngeal cancer, both domestically and internationally. The purpose of this research is to look at the impact, safety, and effectiveness of ERAS in patients undergoing surgery for laryngeal cancer.

## Materials and methods

### Research flow chart

**Figure 1:**
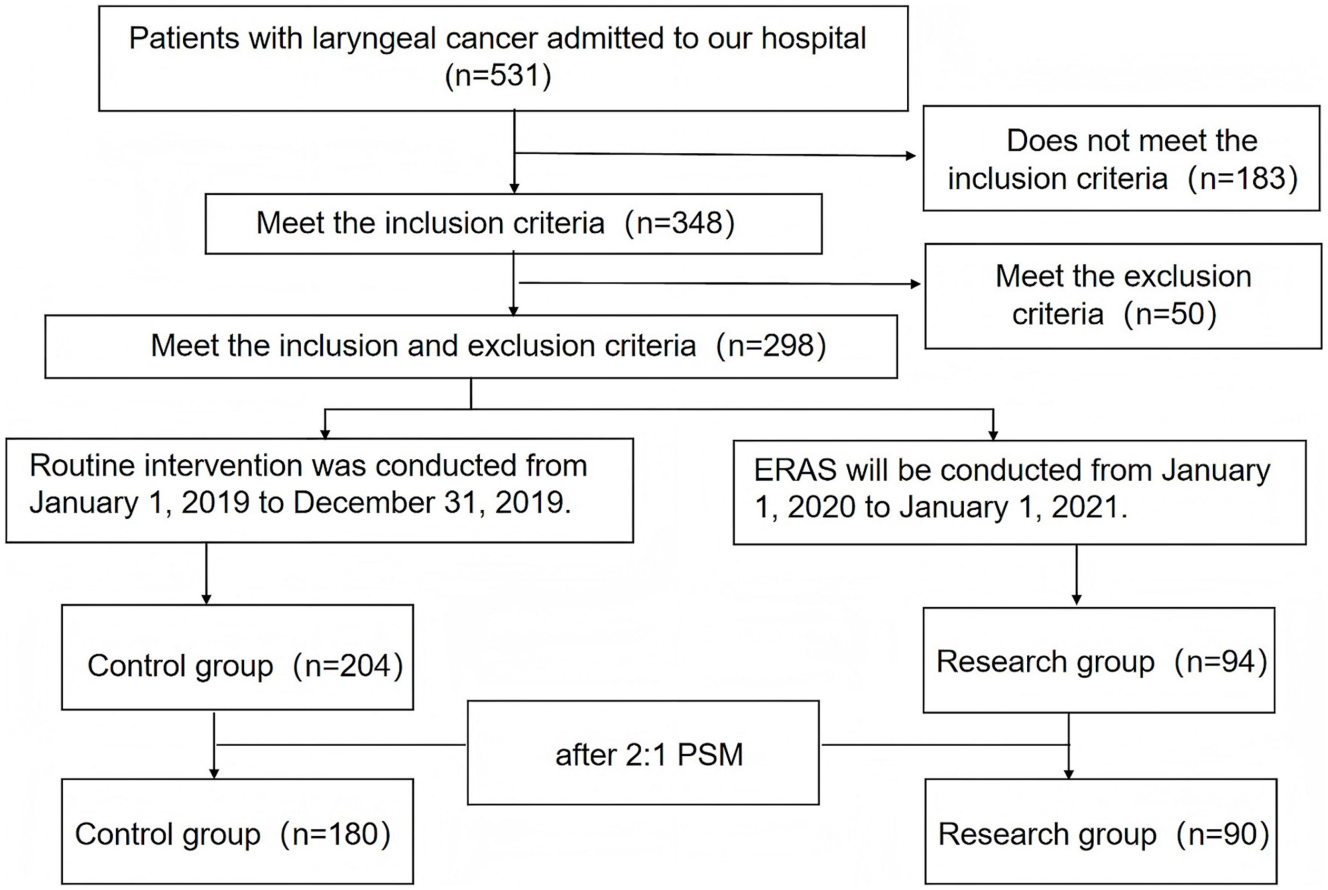
Research flow chart.

Figure 1 shows the flow chart of research.

#### Research objects

The study employed a retrospective cohort design, focusing on patients diagnosed with laryngeal cancer between January 1, 2019, and January 1, 2021, who were scheduled for partial laryngectomy. Individuals accepted between January 1 and December 31, 2019, and receiving standard interventions formed the control group (CG), while those undergoing ERAS from January 1, 2020, to January 1, 2021, comprised the research group (RG). Initially, 531 individuals in all were involved in the trial, with 233 individuals remaining after exclusions (Figure 1). Among them, 94 patients who received ERAS were included in the RG, and 204 patients who received routine intervention were included in the CG. Table 1 shows the comparison of clinical characteristics between the CG and the RG. After matching the propensity score, there were 180 patients in the CG and 90 patients in the RG.

**Table 1:** Comparison of baseline data.

Variable	Before PSM			After PSM		
	Control Group (*n* = 204)	Research group (*n* = 94)	t/χ^2^ and *p*-value	Control Group (*n* = 180)	Research group (*n* = 90)	t/χ^2^ and *p*-value
Age	59.71 ± 9.03	64.70 ± 9.48	−4.35, *p* = 0.000	62.44 ± 9.42	62.93 ± 9.95	0.395, *p* = 0.692
Male	160 (78.43%)	60 (63.83%)	6.36, *p* = 0.012	108 (60.00%)	55 (61.11%)	0.310, *p* = 0.860
Hypertension	87 (42.65%)	39 (41.49%)	0.035, *p* = 0.850	78 (43.33%)	35 (38.89%)	0.487, *p* = 0.570
Diabetes	94 (46.08%)	49 (52.13%)	0.943, *p* = 0.331	89 (49.44%)	48 (53.33%)	0.363, *p* = 0.546
Coronary Heart Disease	68 (33.33%)	34 (36.17%)	0.12, *p* = 0.728	58 (32.22%)	32 (35.56%)	0.300, *p* = 0.583
Albumin(g/L)	40.31 ± 4.53	44.56 ± 4.34	−7.59, *p* = 0.000	44.19 ± 3.44	44.81 ± 2.53	1.516, *p* = 0.130
Prealbumin (mg/L)	240.27 ± 5.15	245.15 ± 3.95	−8.12, *p* = 0.000	245.89 ± 2.45	245.82 ± 4.91	0.156, *p* = 0.875
Education Level			0.814, *p* = 0.665			0.036, *p* = 0.981
Middle school	94 (46.08%)	45 (47.87%)		81 (45.005)	41 (45.56%)	
Senior high school	53 (25.98%)	20 (21.28%)		45 (25.00%)	23 (25.56%)	
University	57 (27.94%)	29 (30.85%)		54 (30.00%)	26 (28.89%)	
Smoking History	66 (32.35%)	30 (31.91%)	0.005, *p* = 0.940	62 (34.44%)	30 (33.33%)	0.033, *p* = 0.855
Surgical Method			1.902, *p* = 0.167			0.007, *p* = 0.931
Partial resection	113 (55.39%)	44 (46.81%)		81 (45.00%)	41 (45.56%)	
Subtotal resection	91 (44.61%)	50 (53.19%)		99 (55.00%)	49 (54.44%)	
Tumor Stage			14.809, *p* = 0.000			0.009, *p* = 0.995
I	57 (27.94%)	19 (20.21%)		38 (21.11%)	19 (21.11%)	
II	114 (55.88%)	41 (43.62%)		75 (41.67%)	38 (42.22%)	
III	33 (16.18%)	34 (36.17%)		67 (37.22%)	33 (36.67%)	

Inclusion criteria:

(1) Patients must have a confirmed diagnosis of laryngeal cancer through clinical and pathological examinations.
(2) Patients undergoing open partial laryngectomy or subtotal laryngectomy, including procedures such as vertical laryngectomy, horizontal laryngectomy, and supracricoid laryngectomy.
(3) Patients must be conscious and capable of cooperating with the study requirements.
(4) Patients should be informed about the study and participate voluntarily.

Exclusion criteria:

(1) Patients who had chemotherapy or radiation before to surgery;
(2) Patients with severe cardiopulmonary diseases or neuromuscular diseases;
(3) Patients with severe mental illness;
(4) Patients with incomplete clinical data;
(5) Patients who were transferred to other hospitals during the study period.

### Intervention method

The CG received routine swallowing rehabilitation training, with a prohibition on swallowing for 7 days post-operation to aid in wound healing. Starting on days 9–10 post-operation, patients were encouraged to undergo oral food training. Initially, nurses guided patients, who still had nasogastric tubes, in attempting to eat, with assistance from caregivers. Adjustments in eating posture were made to find a safe position. Patients showing no significant coughing were introduced to a semi-liquid diet, transitioning to a liquid diet as tolerated. If patients could drink water without coughing, consideration was given to removing the nasogastric tube, enabling normal oral feeding.

Based on the CG, the RG implemented ERAS, and the specific measures are as follows:

(1) Before operation: (1) Comprehensive Patient Evaluation and Treatment Planning: thoroughly assess patients’ conditions to tailor precise treatment plans; actively promote the treatment team and use successful laryngeal surgery cases, postoperative videos, and patient testimonials as educational resources. (2) Psychological intervention: communicate the diagnosis, treatment methods and process of laryngeal cancer positively and compassionately to patients and their families; explain the advantages and specific aspects of surgery offered by the department to build patient trust and confidence, address pre-operative anxiety and fears to bolster patient resolve in facing the disease. (3) Functional training: conduct pre-operative lung function exercises, particularly beneficial for elderly patients; recommend specific exercises such as blowing balloons for 2 h/day, 6 times/day, with each session lasting 20 min, starting 3 days before surgery; advise smoking cessation at least 2 weeks before operation. (4) Pre-operative fasting and hydration: Advise fasting for 6 h before surgery and restrict fluid intake to 2 h before surgery; patients with normal gastrointestinal function and without diabetes can orally consume 800 mL of 12.5% carbohydrate solution 10 h before surgery and 400 mL 2 h before surgery. Diabetic patients should take fructose-containing carbohydrates (e.g., “Delta High” energy supplement liquid, 200 mL/can). (5) Preoperative nutritional assessment: utilize the Nutritional Risk Score 2002 (NRS2002) for pre-operative nutritional assessment; patients identified with severe malnutrition should receive enteral or parenteral nutrition support for 10–14 days before surgery; patients with adequate nutrition may not require pre-operative nutritional support; address pre-operative anemia and hypoproteinemia through interventions such as iron supplementation, blood transfusions, and human albumin administration to optimize nutritional status and reduce complication risks.
(2) During the operation: (1) Ensure patient normothermia (temperature > 36°C) by maintaining room temperature (21–25°C), preheating liquids (≥ 500 mL to ≥36°C), using heating blankets, and other warming devices. (2) Employ elastic stockings and intermittent inflatable compression pumps to provide lower limb massage.
(3) Postoperative position: (1) After the patient returned to the room after the operation, the head of the bed should be appropriately raised by 20° to 35°. (2) Management of various tubes: including tracheal tube, catheter, and nasogastric tube (gastrointestinal decompression tube) care. Tracheal tubes: Replace metal tracheal tubes within 24 h. Gastrointestinal decompression: monitor contents, quantity, color, presence of drainage, and bowel movements within 24 h or on the first day after surgery, ensuring normal gastrointestinal decompression. Catheter: remove catheters 6 h after the bladder fills. (3) Eat early after surgery: On the first day after surgery, initiate enteral nutrition via nasogastric tube, monitor nutritional indicators, and administer nutrients with the following proportion: carbohydrates 45–55%, protein 16–18%, fats 30–35%. Additionally, supplement vitamins, trace elements, and minerals as needed. To achieve the overall calorie needs, provide a daily energy intake of 25–30 kcal/kg and a daily protein intake of 1–2 g/kg. (4) Postoperative mobilization: Immediately after anesthesia, initiate appropriate bed activities such as turning over, knee and ankle extensions, and leg lifts. Encourage family members to massage the patient’s lower limbs. On the first day post-surgery, if the patient shows no discomfort, implement the “three-step bending” method: have the patient sit on the bed for 30 s, then dangle their legs down and sit for another 30 s, followed by standing at the bedside for 30 s. If no adverse symptoms occur, the patient can proceed to get out of bed. Establish activity goals and progressively increase the duration and intensity of activities each day. Aim for at least 2 h of activity on the first postoperative day, increasing to 4–6 h daily until discharge, based on individual patient conditions. Adjustments should be made according to patient-specific considerations. (5) Ensure regular cleaning and disinfection of the inner tube, four times per day (for discharged patients with tubes, instruct patients and their families on post-discharge tube care and disinfection). Emphasize timely and correct sputum aspiration, adhering strictly to aseptic techniques. Perform chest percussion to assist in clearing mucus. Administer intratracheal drops of sodium chloride injection (0.9%) 100 mL, sodium bicarbonate injection 20 mL, and dexamethasone sodium phosphate injection 5 mg twice daily to liquefy sputum, preventing mucus scabbing and potential cannula blockage leading to dyspnea. (6) Swallowing training: On the 6th day post-operation, initiate advanced empty swallowing training, instructing patients to practice short swallows 8–10 times per session, three times daily, for 2–5 days. Based on recovery status, guide patients to attempt to swallow with a stomach tube in place. From 8–10 days after horizontal partial laryngectomy (with mushy or semi-mushy foods like banana or chewed cake), progress to swallowing training. Due to the risk of choking after horizontal partial laryngectomy, ensure patients adopt a specific eating posture: relax, sit upright with a 30-degree head tilt down and chin tucked. Emphasize minimal swallowing attempts.

#### Supraglottic swallowing (autonomous airway protection method)

Prepare food into small balls, instruct patients to inhale, block the tracheal cannula with their index finger, hold their breath, swallow, and then cough spontaneously. This method facilitates smooth food passage into the esophagus while expelling any remaining food in the throat to prevent inhalation.

Mendelssohn Maneuver: Aim to elevate the throat and enhance pharyngeal constrictor strength. Patients with a raised larynx should be guided to put their middle finger on the cricoid cartilage and index finger above the thyroid cartilage in order to feel the larynx lift. They should also be instructed to hold their breath for 2 to 3 s while swallowing and pressing their tongue on the hard palate. Patients with weak throat lifting can receive neck massages to help lift the throat. Ensure patients maintain the lifted position consciously.

Glottic closure training: Focuses on preventing aspiration. Instruct patients to produce a sustained “Yi” sound, transitioning from low to high tones to maximize vocal cord closure.

(1) Observation of patients with horizontal partial laryngectomy using electronic laryngoscope: no significant edema is observed. If the breathing space at the glottic fissure measures more than 0.9 cm (using throat forceps), the tracheal tube can be removed directly. If the space measures less than 0.9 cm, and there is no discomfort such as dyspnea observed 24–48 h post-operation, the tracheal cannula can be removed. Ensure breathing remains smooth during both activity and sleep before sealing the tube.
(2) Physical examination post-operation: assess the wound by palpating and applying pressure to the skin, and observe for any signs of edema and subcutaneous effusion with the assistance of ultrasound.

Adherence to the ERAS protocols was monitored through regular clinical assessments performed by the multidisciplinary team, including anesthesiologists, surgeons, nurses, and nutritionists. Compliance with each ERAS step was recorded in the patient’s medical records, and any deviations from the protocol were documented. Non-compliance was addressed with targeted interventions, such as additional patient education or adjusting the perioperative plan.

### Observation index

#### Baseline data

Patients’ baseline information from the two groups was gathered, including: age, gender, past disease history (including hypertension, diabetes, coronary heart disease, etc.), serum nutritional indicators (albumin, prealbumin), education level (university, senior high school, junior high school), smoking history, surgical methods (partial resection and total resection) and tumor stages (I, II, III and IV).

#### Primary objectives

Hospitalization duration, nasogastric tube and tracheal cannula usage during hospitalization, proportion of enteral nutrition within 24 h post-operation, hospitalization expenses, and readmission rates were recorded for both groups. Additionally, postoperative recovery indices such as complications (subcutaneous effusion, pharyngeal fistula, pulmonary infection) and lower limb venous thrombosis were assessed.

#### Secondary objectives

On the 1st, 3rd, and 7th days after surgery, the nutritional indicators (serum albumin and prealbumin) of both groups were assessed. Both groups’ overall (1-year and 2-year OS) and recurrence-free (1-year and 2-year RFS) survival rates were computed.

#### Propensity score matching

The IBM SPSS program (version 23.0, IBM Corporation, United States) was used to carry out propensity score matching (PSM), and nearest neighbor matching was performed without substitution based on baseline data from control and RG patients, where the caliper value was set to 0.03.

### Statistical analysis

Version 23.0 of IBM SPSS software was used to conduct the statistical analysis (IBM Corporation, United States). Missing data were handled using multiple imputation. The Fisher exact test or the χ^2^ test were used to compare statistical data. When expressing measurement data that followed a normal distribution, ±s, were used, and the T-test was employed for comparison. The measurements represented in M (Q₁, Q₃) did not fit the normal distribution. A Mann–Whitney U test was used to compare groups. A generalized linear model based on a negative binomial distribution was used to compare the frequency of hypoglycemic episodes across the groups. A difference was deemed significant if it was *p* < 0.05.

## Results

### Comparison of baseline data

There wasn’t a noticeable variation in age, sex, hypertension history, diabetes history, coronary heart disease history, albumin level, prealbumin level, education level, smoking history, operation method and tumor stage in the CG (*p* > 0.05; Table 1).

### Main results

#### Postoperative rehabilitation index

**Figure 2:**
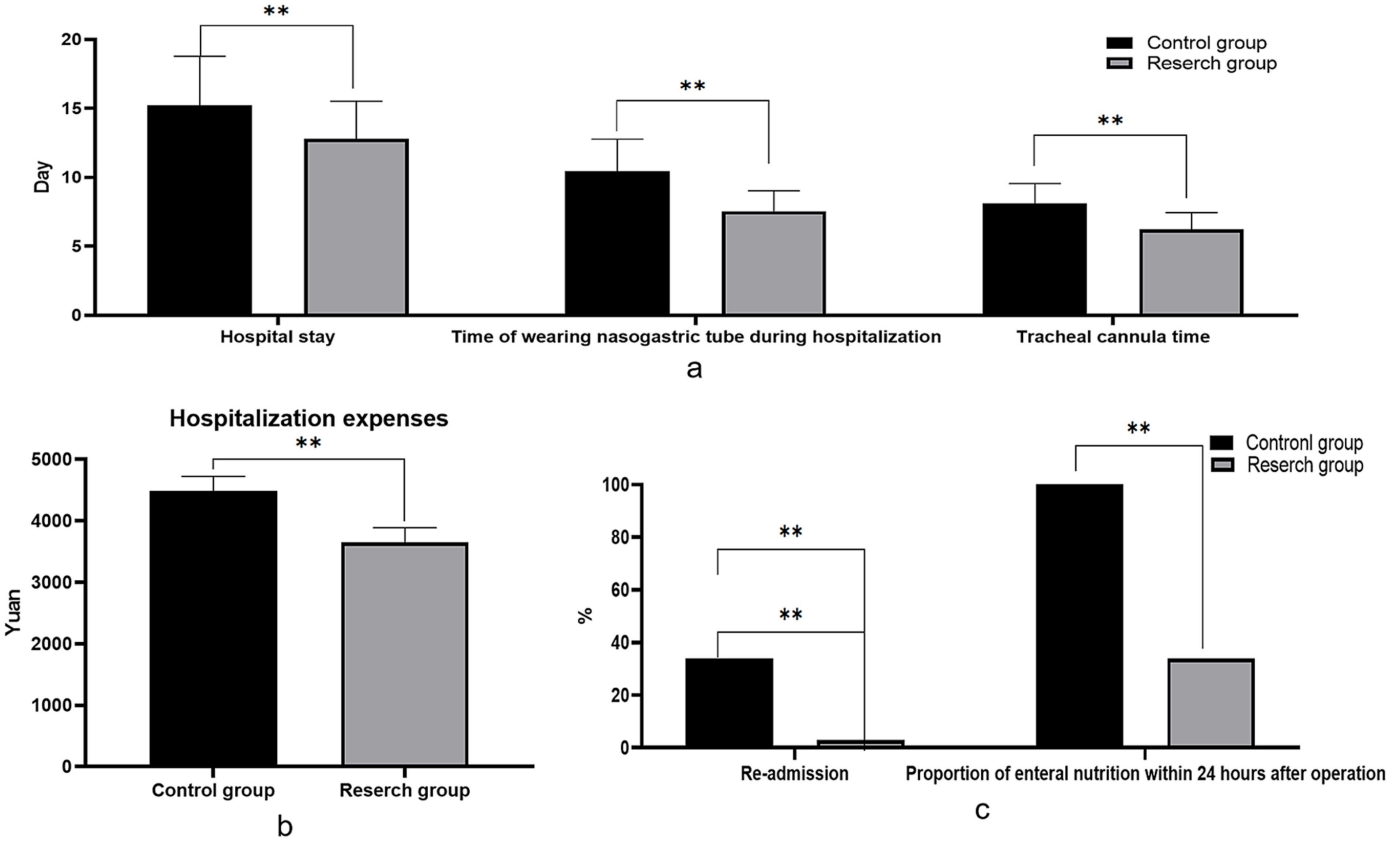
Comparison of postoperative rehabilitation indexes between the two groups. **(A)** The length of hospitalization, the time of wearing nasogastric tube and the time of tracheal tube during hospitalization were compared involving the two groupings; **(B)** Comparison of hospitalization costs involving the two groupings; **(C)** Proportion of enteral nutrition and readmission rate within 24 h after surgery in both groups.

The hospital stay, the time of wearing nasogastric tube and tracheal cannula, the proportion of enteral nutrition within 24 h after operation, hospitalization expenses and readmission rate of the two groups were considerably lower in the RG than in the CG (*p* < 0.05; Figure 2).

#### Postoperative complications

Compared with the CG, the incidence of postoperative complications in the RG was lower than that in the CG, and the two groups did not vary statistically from one another. Both groups had patients who experienced language disorders; however, there was no significant difference between the groups in terms of the incidence of language disorders (*p* > 0.05). All patients with language disorders received speech rehabilitation therapy until their condition improved and they were discharged from the hospital (*p* > 0.05; Table 2).

**Table 2:** Comparison of postoperative complications.

Variable	Control group (*n* = 180)	Research group (*n* = 90)	χ^2^ and *p*-value
Subcutaneous effusion	1 (0.56%)	0	0.125, *p* = 0.723
Pharyngeal fistula	2 (1.11%)	0	0.063, *p* = 0.801
lung infection	2 (1.11%)	0	0.063, *p* = 0.801
Venous thrombosis of lower extremity	1 (0.56%)	0	0.125, *p* = 0.723
Language barrier	2 (1.11%)	1 (1.11%)	0.0000, *p* = 1.000
Total	8 (4.45%)	1 (1.11%)	2.069, *p* = 0.150

### Secondary result

#### Nutritional index

The levels of albumin and prealbumin in the RG on the 3rd and 7th postoperative day were considerably higher than those in the CG (*p* < 0.05). There was no difference in albumin level on the 1st day after operation (*p* > 0.05; Table 3).

**Table 3:** Comparison of nutritional indicators.

Variable	After PSM control group (*n* = 180)	Research group (*n* = 90)	t and *p*-value
Serum albumin			
The first postoperative day	39.85 ± 4.91	40.82 ± 2.75	1.741, *p* = 0.082
The third postoperative day	35.81 ± 3.91	39.71 ± 2.81	8.432, *p* = 0.000
The seventh postoperative day	37.28 ± 3.55	40.59 ± 2.71	7.781, *p* = 0.000
Prealbumin			
The first postoperative day	230.81 ± 2.01	241.81 ± 3.91	30.556, *p* = 0.000
The third postoperative day	164.18 ± 2.85	206.84 ± 3.85	102.725, *p* = 0.000
The seventh postoperative day	187.48 ± 2.85	231.84 ± 4.91	93.758, *p* = 0.000

#### Overall survival rate and recurrence-free survival rate

**Figure 3:**
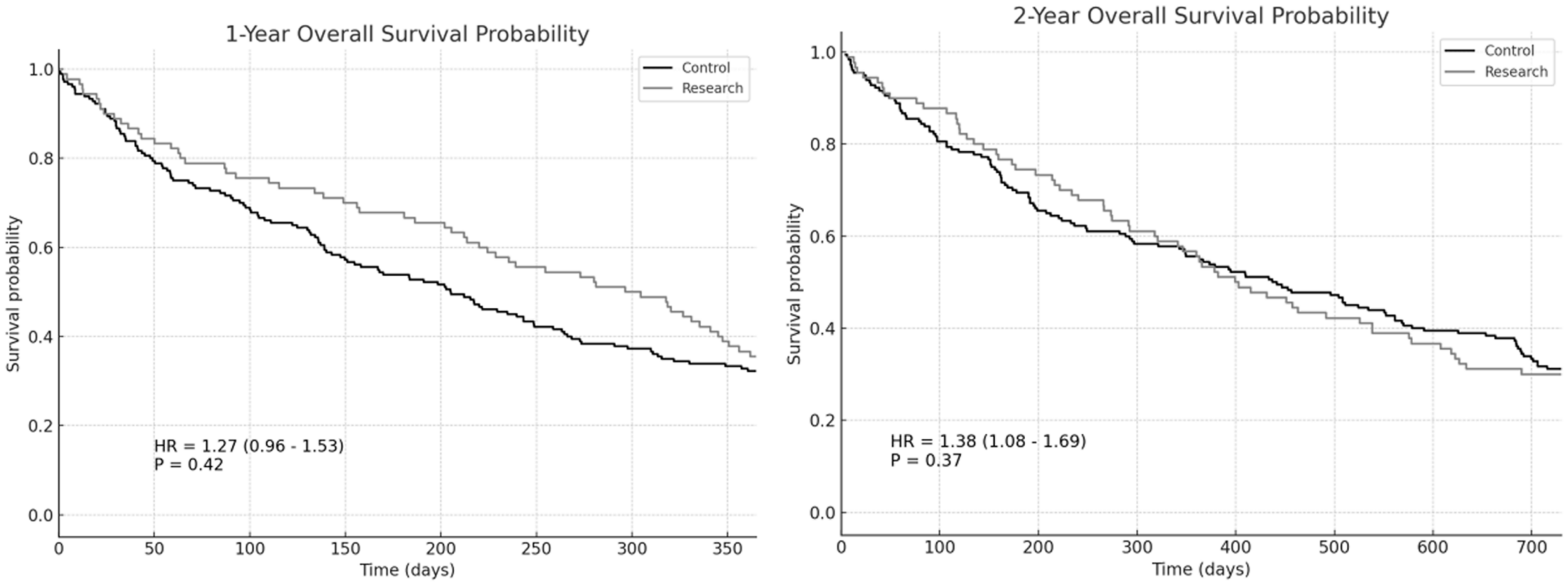
The 1-year and 2-year overall survival rates of the two groups.

**Figure 4:**
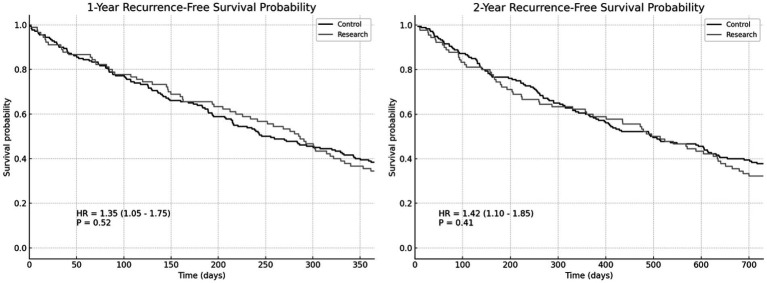
The 1-year and 2-year recurrence-free survival rates of the two groups.

There wasn’t a noticeable variation in 1-year overall survival rate involving the two groupings (HR = 1.27, *p* = 0.42). There wasn’t a noticeable variation in 2-year overall survival rate (HR = 1.38, *p* = 0.37). There was no significant difference in 1-year recurrence-free survival rate involving the two groupings (HR = 1.35, *p* = 0.52). There wasn’t a noticeable variation in 2-year recurrence-free survival rate involving the two groupings (HR = 1.42, *p* = 0.41). However, the survival rate of the RG is slightly better than that of the CG (Figures 3, 4).

## Discussion

The throat has important physiological functions such as pronunciation, breathing and swallowing (6–8). Patients undergoing surgery may experience partial or complete loss of pronunciation function, necessitating long-term tube use and a stoma on the body surface. These outcomes significantly impact physical appearance, physiological function, mental health, and daily social activities (9). Such conditions can lead to social stigma, evoke negative psychological reactions, and contribute to feelings of anxiety and depression among patients (10). Cancer-related negative emotions will have a harmful effect on patients’ treatment compliance and quality of life, resulting in a 10–20% decline in overall survival rate (11). Patients need to adopt different strategies to deal with the threat of the disease itself and the direct and long-term effects of surgical treatment. Nursing staff intervention should commence preoperatively and continue throughout the entire process to assist patients in stabilizing their emotions (12, 13). This approach aims to alleviate the inferiority complex stemming from their condition, mitigate the anxiety and stress commonly associated with cancer, bolster patients’ confidence in actively managing their illness, and foster better cooperation with treatment and nursing care (14). The gradual popularity of ERAS concept at home and abroad has changed the clinical treatment mode of many diseases. The concept of accelerated rehabilitation surgery for laryngeal cancer runs through the whole process before, during and after the operation. Through various effective measures, the highest quality nursing is combined with the most advanced technology to reduce the stress response of patients, promote wound healing and restore organ function, thus reducing the incidence of postoperative complications. ERAS is a humanized perioperative nursing concept centered on patients everywhere (14, 15). Based on this, this study explored the influence of ERAS on postoperative rehabilitation, complications and long-term prognosis of patients undergoing radical laryngectomy. However, this study was retrospective and unable to control for confounding factors through random grouping as in prospective studies. Therefore, the study utilized the Propensity Score Matching (PSM) method to balance baseline differences involving the two groupings. PSM is a statistical technique primarily employed for subgroup analysis in observational clinical research or clinical trial data. It effectively mitigates confounding biases, aiming to approximate results similar to those obtained from randomized controlled studies (16–18).

Combined with the results of this study, the hospital stay, the time of wearing nasogastric tube and tracheal cannula, the proportion of enteral nutrition within 24 h after operation, hospitalization expenses and readmission rate of the two groups were lower than those of the CG. Compared with the CG, the incidence of postoperative complications in the RG was lower than that in the CG, and the two groups did not vary statistically. The application of ERAS concept in radical laryngectomy can significantly shorten the hospitalization time of patients, reduce the wearing time of nasogastric tube and tracheal cannula, improve the proportion of early postoperative enteral nutrition, reduce hospitalization expenses, and reduce the rate of readmission. These results indicate that the ERAS concept enhances the speed of postoperative rehabilitation, reduces the economic burden on patients, and provides substantial benefits. The incidence of postoperative complications did not show a statistically significant difference between the RG and the CG. Remarkably, the frequency of complications in the RG was lower than that in the CG. This suggests that the ERAS approach effectively mitigated the risk of complications through comprehensive measures such as preoperative education, precise intraoperative management, and early postoperative rehabilitation exercises.

The levels of albumin and prealbumin in the RG on the 3rd and 7th postoperative day were higher than those in the CG. There was no difference in albumin level on the 1st day after operation. There wasn’t a noticeable variation in 1-year overall survival rate involving the two groupings (HR = 1.27, *p* = 0.42). There wasn’t a noticeable variation in 2-year overall survival rate (HR = 1.38, *p* = 0.37). There wasn’t a noticeable variation in 1-year recurrence-free survival rate involving the two groupings (HR = 1.35, *p* = 0.52). There wasn’t a noticeable variation in 2-year recurrence-free survival rate between the two groups (HR = 1.42, *p* = 0.41). However, the survival rate of the RG is slightly better than that of the CG. The application of the ERAS concept in patients undergoing radical laryngectomy significantly enhances postoperative nutritional status, potentially positively impacting long-term survival rates. Improved nutritional status is crucial for accelerating recovery post-operation. Albumin and prealbumin levels serve as vital indicators of patients’ nutritional status, with increased levels indicating improved nutritional intake and enhanced metabolic function (19). The individuals in the RG showed better nutritional status in the early postoperative period, which may be related to the early postoperative enteral nutrition support emphasized in ERAS. Through early and adequate nutritional intake, patients can expedite their recovery from surgery, shorten recovery times, and enhance their overall quality of life. Patients in the RG had marginally higher survival rates than those in the CG, despite the fact that there was no statistically significant difference between the two groups’ overall survival rate and recurrence-free survival rate in this investigation. This finding suggests that the ERAS concept may positively influence long-term patient survival by improving nutritional status, reducing complications, and supporting postoperative rehabilitation. Our study highlights several key components of the ERAS protocol that contributed significantly to improved outcomes, particularly early mobilization, early enteral nutrition, and optimized pain management. Early mobilization has been shown to reduce hospital stay, enhance functional recovery, and decrease complications such as venous thromboembolism and pneumonia. Additionally, effective pain management strategies, including the use of multimodal analgesia, helped reduce opioid use, minimizing opioid-related side effects and facilitating faster recovery. These components of ERAS work synergistically to reduce hospital stay and complications, ultimately improving patient outcomes. In line with recent studies comparing primary surgery and radiochemotherapy for locally advanced laryngeal cancer, such as the study by Shelan et al. (20), primary surgery followed by adjuvant treatments is associated with superior loco-regional control (LRC) compared to primary chemoradiotherapy (CRT). Although our study focuses on the implementation of the ERAS protocol in the surgical context, it is worth noting that the success of surgical treatment, when paired with early postoperative rehabilitation, likely contributes to better recovery and improved overall outcomes. This finding is consistent with our results, where ERAS patients showed reduced recovery times and fewer complications compared to controls. However, the OS rates between surgery and CRT did not differ significantly, highlighting the need for further research into the ideal treatment strategies for patients with advanced-stage laryngeal cancer.

While our study demonstrates statistically significant improvements in postoperative recovery and complication rates with the ERAS protocol, it is also important to consider the clinical significance of these findings. For instance, the reduction in hospital stay, the duration of nasogastric tube use, and the faster recovery to normal eating patterns directly impact patient comfort and quality of life. These clinically meaningful changes in recovery time and complication rates not only enhance the patient experience but also reduce healthcare burdens. ERAS has the potential to improve patient satisfaction and decrease the likelihood of long-term complications, which are key indicators of successful recovery. However, one limitation of our study is the relatively short follow-up period for cancer outcomes, particularly (OS and RFS). Although we observed marginal differences in survival rates between the ERAS and control groups, the short follow-up (1–2 years) may not fully capture the long-term benefits or risks of the ERAS protocol. Longer follow-up is required to assess whether the early postoperative recovery benefits translate into improved long-term cancer outcomes. Implementing the ERAS protocol presents several challenges, particularly in ensuring multidisciplinary collaboration and adherence to standardized care pathways. A key barrier is the integration of ERAS into the clinical routine, which requires active involvement from various healthcare providers, including surgeons, anesthesiologists, nurses, and nutritionists. Additionally, institutions with limited resources or those with varying levels of ERAS adoption may encounter difficulties in fully implementing these protocols. Despite these challenges, our study demonstrates the feasibility of implementing ERAS in a single-center setting, and future studies should explore strategies for overcoming these barriers and scaling ERAS protocols to broader, multi-center environments. Additionally, our study did not analyze the effects of ERAS on specific subgroups, such as patients categorized by age, cancer stage, or comorbidities. Certain subgroups, such as older patients or those with multiple comorbidities, may derive more significant benefits from the early postoperative recovery strategies within the ERAS protocol. Future research should explore these subgroup effects to tailor ERAS more effectively to individual patient needs. Despite our efforts to minimize baseline differences using PSM, several potential confounders remain, including patient comorbidities (e.g., hypertension, diabetes), variations in surgical techniques (partial vs. subtotal laryngectomy), and the time-based cohort allocation (2019 vs. 2020). The retrospective, single-center design of our study also limits the generalizability of the results. Moreover, temporal bias introduced by changes in clinical practices or external factors (e.g., COVID-19) during the study period may have influenced outcomes. Although PSM was applied to address some of these confounders, further multicenter, prospective studies are necessary to better control for these variables, confirm the generalizability of our findings, and validate the long-term effectiveness of ERAS in postoperative rehabilitation for laryngeal cancer patients.

## Conclusion

The implementation of ERAS treatment and nursing interventions in postoperative patients with laryngeal cancer has demonstrated several significant benefits. These include effectively reducing hospitalization days, minimizing the duration of nasogastric tube and tracheal cannula use, lowering hospitalization costs, decreasing readmission rates, mitigating postoperative complications, accelerating recovery, and facilitating early patient discharge. The application of accelerated rehabilitation surgery concepts in the perioperative period of laryngeal cancer patients promotes swift recovery, enhances patient comfort, and yields positive clinical outcomes, thus warranting further clinical promotion and adoption.

## Data Availability

The original contributions presented in the study are included in the article/supplementary material, further inquiries can be directed to the corresponding author.
